# Assessing Risk-Based Policies for Pretrial Release and Split Sentencing in Los Angeles County Jails

**DOI:** 10.1371/journal.pone.0144967

**Published:** 2015-12-29

**Authors:** Mericcan Usta, Lawrence M. Wein

**Affiliations:** 1 Management Science & Engineering Department, Stanford University, Stanford, CA, United States of America; 2 Graduate School of Business, Stanford University, Stanford, CA, United States of America; British Columbia Centre for Excellence in HIV/AIDS, CANADA

## Abstract

Court-mandated downsizing of the CA prison system has led to a redistribution of detainees from prisons to CA county jails, and subsequent jail overcrowding. Using data that is representative of the LA County jail system, we build a mathematical model that tracks the flow of individuals during arraignment, pretrial release or detention, case disposition, jail sentence, and possible recidivism during pretrial release, after a failure to appear in court, during non-felony probation and during felony supervision. We assess 64 joint pretrial release and split-sentencing (where low-level felon sentences are split between jail time and mandatory supervision) policies that are based on the type of charge (felony or non-felony) and the risk category as determined by the CA Static Risk Assessment tool, and compare their performance to that of the policy LA County used in early 2014, before split sentencing was in use. In our model, policies that offer split sentences to all low-level felons optimize the key tradeoff between public safety and jail congestion by, e.g., simultaneously reducing the rearrest rate by 7% and the mean jail population by 20% relative to the policy LA County used in 2014. The effectiveness of split sentencing is due to two facts: (i) convicted felony offenders comprised ≈ 45% of LA County’s jail population in 2014, and (ii) compared to pretrial release, split sentencing exposes offenders to much less time under recidivism risk per saved jail day.

## Introduction

To mitigate severe prison overcrowding, the U.S. Supreme Court (*Brown v. Plata*, 2011) forced the state of California (CA) to reduce its prison population by 25% within two years. In response, CA passed the Public Safety Realignment Act (Assembly Bill 109, often referred to as realignment), which called for low-level (i.e., non-serious, non-violent, non-sex-related) felonies and state parole violations to be punishable by incarceration in county jails rather than state prisons. Although realignment has successfully reduced the state prison population, it has caused a significant increase in the CA jail population: of the 58 CA counties, 19 (including LA County) have court-ordered jail population caps [1] (some counties rent jail space from other counties), and 21 counties are receiving CA state funds to add more than 10,000 additional jail beds [2].

CA counties have two primary options for reducing jail overcrowding in the short run. They can offer pretrial release to defendants, in the hope that these defendants appear in court and do not recidivate (i.e., commit another crime) prior to case disposition. In addition, Assembly Bill 1468 requires that—unless the court finds it is not in the interest of justice—as of January 1, 2015 low-level felony sentences be split between jail time and mandatory supervision. To aid in these decisions, correctional systems throughout the U.S. employ risk-based tools that use a defendant’s demographic data and criminal history to predict the likelihood of recidivism and of appearing in court. These tools are moderately predictive, achieving an area under the curve of the receiver operating characteristic curve of ≈ 0.7 [3], meaning, e.g., that the probability a three-year recidivist has a higher risk score than a three-year non-recidivist is 0.7.

To investigate jail management under these circumstances, we build a simulation model that tracks the flow of inmates over time in LA County jails, which is the world’s largest jail system. In our model, individuals arrive for arraignment as one of six types, according to whether their current charge is a felony or non-felony, and whether their risk category is low, medium or high in the California Static Risk Assessment (CSRA) tool [4], which is one of the risk tools used in CA. Using a queueing network model for process flow and statistical models for risk-based recidivism and failure to appear in court, we follow individuals through arraignment, pretrial detention or release, case disposition and jail sentence, as well as recidivism that may occur during pretrial release, after a failure to appear in court, during regular probation for non-felonies, and during supervision of a split sentence for felonies. We assess 64 joint pretrial release and split-sentencing policies that are risk-based, and compare them to the status quo policy that LA County was using in 2014; despite the passage of Assembly Bill 1468, LA County used split sentencing only sparingly in early 2015 (Fig 6 in [5]). Our goal is to identify policies that optimize the tradeoff between public safety—as measured by the annual rearrest rate of anyone on pretrial release, after a failure to appear in court, on regular probation or on supervision during a split sentence—and jail congestion, as measured by the mean jail population or the mean amount of jail overcrowding (i.e., population in excess of jail capacity).

## Methods

**Fig 1:**
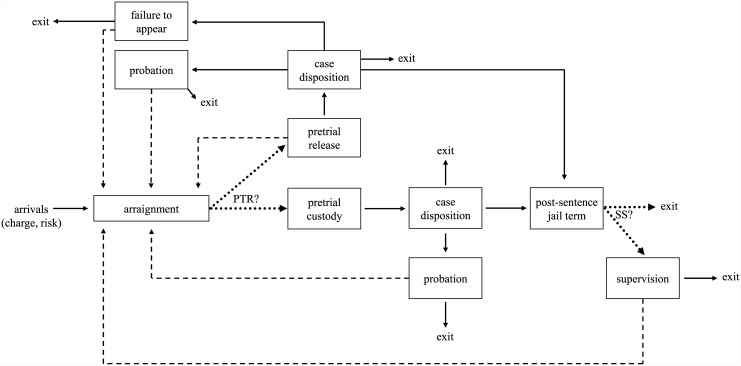
A depiction of the process flow. The two key decisions (dotted lines) are whether to offer pretrial release (denoted by PTR?) and split sentencing (SS?), where the latter is available only to felons. The key tradeoff is between public safety, as measured by recidivism (dashed lines), and jail population, which is the total number of inmates waiting for arraignment, in pretrial custody or serving a post-sentence jail term. Each arrival has a charge type (non-felony or felony) and a CSRA risk category (low, medium or high), and some of the routing probabilities and time durations are functions of charge type, risk category and/or pretrial status (release or custody).

The model is depicted in Fig 1, the policies are described in Tables 1 and 2, and a list of model parameters and their values are given in Tables 3 and 4. Details of the parameter estimation procedure appear in S1 File.

**Table 1:** The 64 policies are all combinations of one option from each of the three columns. The numbers in the pretrial release columns are used in Fig 3 to refer to these policies.

Pretrial Release for Non-felony	Pretrial Release for Felony	Split-sentencing for Felony
0—no one	0—no one	no one
1—only low risk	1—only low risk	only low risk
2—low and medium risk	2—low and medium risk	low and medium risk
3—everyone	3—everyone	everyone

**Table 2:** The pretrial release probabilities for the status quo policy in LA County (see §B in S1 File for derivations). The status quo policy does not offer split-sentencing to any offenders.

Charge	Risk	Probability of Pretrial Release
non-felony	low	0.80
non-felony	medium	0.70
non-felony	high	0.60
felony	low	0.55
felony	medium	0.31
felony	high	0.10

**Table 3:** Pre-disposition parameters and values for defendants. The two lognormal parameters are the mean and standard deviation of the underlying normal distribution.

Parameter Description	Parameter Value	Reference
jail capacity	19,000	[11, 12]
interarrival times (days)	Erlang(shape = 2, scale = 1/395)	[13], §C in S1 File
time delay until arraignment	lognormal(3.87,0.51)	[6], §D in S1 File
proportion non-felony	0.558	[6]
proportion felony	0.442	[6]
proportion low risk	0.170	[15]
proportion medium risk	0.281	[15]
proportion high risk	0.549	[15]
time to recidivism, low risk	∞ with probability 0.23 otherwise, lognormal(0.94,2.80)	[15], §E in S1 File
time to recidivism, medium risk	∞ with probability 0.23 otherwise, lognormal(0.14,1.98)	[15], §E in S1 File
time to recidivism, high risk	∞ with probability 0.23 otherwise, lognormal(−0.64,1.16)	[15], §E in S1 File
failure to appear, low risk	0.117	[17], §F in S1 File
failure to appear, medium risk	0.178	[17], §F in S1 File
failure to appear, high risk	0.178	[17], §F in S1 File

**Table 4:** Parameters and values related to case disposition. The gamma parameters are the shape and the scale. The lognormal parameters are the mean and standard deviation of the underlying normal distribution. The triangular parameters are the minimum, the maximum, and the mode.

Description	Non-felony		Felony		Reference
	Pretrial Release	Pretrial Custody	Pretrial Release	Pretrial Custody	
time from arraignment to disposition (days)	gamma (1.07,119.78)	gamma (0.46,16.80)	lognormal (5.13,0.47)	gamma (0.67,76.81)	[6] §G in S1 File
proportion dismissed	0.207	0.052	0.153	0.069	[6] §H in S1 File
proportion in probation	0.481	0.280	0.681	0.534	[6, 21, 22] §H in S1 File
proportion in jail (and (probation if non-felony)	0.312	0.668	0.066	0.208	[6, 21, 22] §H in S1 File
proportion in prison	—	—	0.100	0.189	[6, 21, 22] §H in S1 File
post-sentence jail term (days if non-felony, months if felony)	gamma (0.368,29.92)	gamma (0.397,77.08)	lognormal (1.203,0.633)	lognormal (2.064,0.628)	[6, 23] §I in S1 File
length of probation (years)	triangular (0,3,1)	triangular (0,3,1)	triangular (1,5,3)	triangular (1,5,3)	[24, 25]

### Process Flow

New inmates arrive to the county jail system according to a renewal process, where the time between consecutive arrivals has an Erlang distribution. The county jail has a fixed capacity, but we assume that some detainees are held in a different jail (e.g., in another county or at a U.S. Immigration and Customs Enforcement facility) if the current jail population exceeds its capacity. The arriving defendants are randomly assigned to one of six types, according to a combination of their charge (non-felony or felony, where the former consists of misdemeanors and lesser charges) and their CSRA risk category (low, medium or high), where the risk category and charge probabilities are assumed to be statistically independent. After a short random delay, defendants undergo arraignment, during which the first of two key decisions is made: based on a defendant’s charge-risk type, either release the defendant until case disposition (i.e., pretrial release) or hold him (we adopt the male gender throughout) in custody until case disposition (our model does not incorporate the many arrests that do not result in arraignment [6]).

The time delay from arraignment until case disposition is random and depends upon a defendant’s charge (non-felony vs. felony) and pretrial release vs. custody status, but not on his CSRA risk. Defendants on pretrial release possess two competing random times: the time from arraignment to recidivism (which is based on a statistical model that depends on his CSRA risk but not on his charge, and which can be infinite) and the time from arraignment until case disposition. If the former time is shorter than the latter time, then the defendant recidivates before case disposition; his recidivism charge is assumed to be the same as his original charge and his risk is unchanged (note that CSRA and some other risk tools do not use the current charge as a predictive variable due to its lack of predictiveness [4]). The recidivating defendant re-enters the jail and waits for a new arraignment, and the new pretrial release vs. custody decision takes into account his recent recidivism, as described later.

If the time from arraignment to case disposition is shorter than the time from arraignment to recidivism for a defendant on pretrial release, he does not recidivate before case disposition. In this case, we assume that the likelihood of failing to appear in court for case disposition depends on the defendant’s risk category but not on his charge type. If he does not appear in court, then his time from arraignment to recidivism remains active, and he may recidivate at a later time, at which point he is treated in the same way as those who recidivate before case disposition.

Case disposition for non-felonies has three possible outcomes, with probabilities that depend upon the pretrial release vs. custody status: acquittal/dismissal (and exit from the system), probation, or a jail term that also includes a probation component. The random length of probation is statistically identical, whether or not it is preceded by a jail term. Similarly, felony cases have four possible dispositions with probabilities that depend upon the release vs. custody status: acquittal/dismissal, probation, jail (without probation), and prison, where those going to prison exit our model. The length of the post-sentence jail term depends on both the charge (non-felony vs. felony) and the pretrial status (release vs. custody). Later we discuss the key assumption that the time from arraignment to case disposition, the court outcome and the length of the post-sentencing jail term depend on whether the offender is released or detained prior to trial.

The second of our two policy decisions is made during case disposition of felonies: whether or not—depending upon the risk category of the offender—to offer split sentences for felonies (all felons in our model are low-level felons that are eligible for split sentencing). Felons receiving a split sentence spend the first half of their post-sentence jail term in jail, and spend the second half out on mandatory community supervision, where the 50–50 split is based on recent reports from CA counties [5, 7, 8]. Finally, offenders on probation or supervision are assumed to recidivate according to the same statistical model as offenders on pretrial release, but—in contrast to recidivists on pretrial release, who are typically released for a shorter amount of time—they are assigned a new charge at random (although their risk does not change) before returning for re-arraignment, and the new pretrial release vs. custody decision takes into account his recent recidivism, as described later.

### Policies

Our pretrial release decisions are based on an offender’s charge-risk type, and the split-sentencing decisions for felons are based on their risk category. We restrict ourselves to policies that are independent of the current number of inmates in jail and are monotonic in risk; i.e., if a certain offender is offered pretrial release then all offenders with the same charge and with the same or lower risk is also offered pretrial release, and if a felon is offered a split sentence then all felons with the same or lower risk is also offered a split sentence. Hence, because there are four options for each risk category (offering the option to no one, to only low-risk individuals, to low- and medium-risk individuals, or to everyone) and three decisions (pretrial release for non-felonies, pretrial release for felonies, split-sentencing for felonies), we consider 4^3^ = 64 policies that correspond to all combinations of one option from each of the three columns in Table 1.

In addition, we consider a policy that represents the status quo in LA County in early 2014. This policy does not offer split-sentencing to any offenders because LA County’s use of split sentencing was < 1% during June 2013—May 2014 (Fig 6 in [5]). The probability of pretrial release in LA County depended upon the charge-risk type (Table 2), and these probabilities are estimated using data in [6, 9, 10] (§A in S1 File).

The decisions in Tables 1 and 2 apply only to new arrivals. If an offender recidivates during pretrial release, then he is detained after rearraignment under the 64 policies in Table 1 and the status quo policy. If an offender recidivates during probation or supervision, then he is offered pretrial release with probability 0.2 if his recidivism charge is a non-felony and with probability 0.1 if his recidivism charge is a felony, independent of an offender’s risk category (§B in S1 File); these pretrial release probabilities are based on financial conditions (i.e., defendants are able to post bail or a bond, which is rarely denied in LA County) rather than risk, and are applied to all 65 policies.

### Performance Measures

Our key tradeoff is between public safety and jail congestion. We measure public safety by the annual number of jailed rearrests of (i.e., recidivist crimes by) anyone on pretrial release, probation or supervision, or after a failure to appear in court. We measure jail congestion in two ways: by the mean size of the jail population over the length of the simulation, or by the mean amount of overcrowding, which is the mean of the number of inmates in excess of the county jail capacity at each point in time.

### Parameter Estimation

#### Jail Capacity

The jail capacity of 19,000 is approximately halfway between the functional bed capacity (set at 90% of capacity to allow for seasonal fluctuations and the need to separate special-need and high-risk inmates) of LA County projection options A and C (19,530) and option B (18,630) in Table 14 of [11]. This estimate is also consistent with the rated (by the Board of State and Community Corrections) capacity with the inclusion of fire camps of 19,474 (page 4 of [12]) minus the ≈ 500 prison inmates and transfers that are housed in jail.

#### Interarrival Times

The interarrival time distribution is derived from arraignment data (e.g., [13]) during 2008–2012 in LA County (§C in S1 File).

#### Time Delay From Arrest To Arraignment

The parameters for the time delay from arrest to arraignment are derived from 2008 data from LA County [6] via maximum likelihood estimation (§D in S1 File).

#### Charge Proportion

Using the “Cases Matched from PIMS to AJIS” column in Table 3 of [6], we estimate that the proportion of defendants who have a felony charge is 49,549/112,201 = 0.442, and the proportion who have a non-felony charge is 0.558.

#### Risk Tool

We initially considered two risk tools, Correctional Offender Management Profiling for Alternative Sanctions (COMPAS) [14] and CSRA [4], that have been used by CA correctional agencies and externally validated (albeit on pre-alignment state parole populations rather than post-alignment jail populations). The main advantage of COMPAS is that it has a finer granularity of risk (10 risk categories) than CSRA (three risk categories). However, we chose to adopt CSRA in this study because its validation study for recidivism [15] has finer temporal granularity and less right-censoring (recidivism at 1, 2 and 3 years for 110,313 parolees) than the COMPAS validation study [16] (recidivism at 2 years for a sample of 24,418 parolees), both of which are required to develop a reliable statistical model for time to recidivism. Here, recidivism refers to an arrest and return to custody, which is the most relevant definition for jail capacity and cost [14]. However, because we could not locate any studies that calibrated the CSRA risk tool to failure-to-appear data, we use COMPAS (after aggregating its 10 risk categories into CSRA’s three risk categories, as in page 20 in [17]) to estimate the risk-based likelihood of failing to appear in court.

#### Risk Proportion

We use the risk category breakdown of the 110,313 parolees in Table 15 of [15] to get the proportions in Table 3.

#### Time to Recidivism

Using maximum likelihood estimation, we fit five models (§E in S1 File) to the raw data in Table 15 of [15] (recidivism within 1 year, 2 years and 3 years for each of three risk categories): a lognormal model (where the mean parameter is an affine function of the risk score), a split lognormal model (where a proportion of the population—independent of risk—never recidivate [18] and the others recidivate according to a lognormal distribution), a split lognormal model with heteroskedasticity (the standard deviation parameter is also an affine function of the risk score), a proportional hazards model [19], and a split proportional hazards model. The split lognormal model with heteroskedasticity provides the best fit (§E in S1 File). The lognormal distribution exhibits a unimodal hazard rate, which captures a brief period of desistance followed by a peak incidence of recidivism and a slowdown thereafter (Fig A in S1 File). The split improves the empirical fit, as seen in earlier studies (e.g., [20])

#### Failure to Appear

We use a study that validates the COMPAS tool using 18 months of data from Broward County’s (FL) jail system [17] to estimate the failure-to-appear probabilities in Table 3 (§F in S1 File).

#### Time from Arraignment to Disposition

We use maximum likelihood estimation to fit lognormal and gamma distributions to arrest-to-disposition time data from [6] (§G in S1 File).

#### Case Disposition Probabilities

The 14 case disposition probabilities in Table 4 are estimated in §H in S1 File using Bayes rule and data in Table 4 of [6], pages 57 and 129 of [6] and Table 1 in [21], and odds ratio estimates on page 10 of [22].

#### Post-sentence Jail Terms

We fit mixture (of pretrial release and pretrial custody) lognormal and gamma distributions for jail sentences using non-felony data on page 129 in [6] and Fig 25 in Appendix C in [6], and felony data in Chart 3 in [23] (§I in S1 File).

#### Length of Probation

Although typical non-felony probation duration is widely reported by criminal law offices as one year (with no minimum and a maximum of three years) [24] and typical felony probation duration is given as three years (with a minimum of one year and a maximum of five years) [25], we could not locate data on their distribution. Consequently, we chose triangular distributions with these ranges and with modes as their typical durations.

## Results

For all reported results, we simulate 1000 runs of 2000 days each—collecting statistics only after the 900^th^ day—and report on the mean of the 1000 replications.

### Model Validation of Jail Population and Composition

We begin by simulating the status quo policy (Table 2) and find that the total jail population, the composition of felons vs. non-felons and sentenced vs. non-sentenced, and the amount of overcrowding are generally consistent with reported values for LA County (Table 5).

**Table 5:** Model validation of the status quo policy.

Statistic	Simulation of Status Quo Policy	Reported Values
mean jail population	17,744 ±48	18,693 in 2013 [43] 17,712 in Oct. 2012 [44] 16,448 in Feb. 2012 [11]
felons in jail	14,026 ±43 (79%)	17,259 (0.923) in 2013 [43] 0.78 in Dec. 2011 [11]
non-felons in jail	3718 ±13 (21%)	1434 (0.077) in 2013 [43] 0.22 in Dec. 2011 [11]
sentenced inmates	10,182 ±35 (57.4%)	8845 (0.473) in 2013 [43] 8378 (0.451) in June 2013 [45] 0.55 in Dec. 2011 [11]
non-sentenced inmates	7562 ±19 (42.6%)	9848 (0.527) in 2013 [43] 10,198 (0.549) in June 2013 [45] 0.45 in Dec. 2011 [11]
fraction of time overcrowded	0.151 ±0.009	0.132 in 2012 [13, 43]

### A Simple Metric

To provide a framework for interpreting our main results, we introduce a simple metric that quantifies the tradeoff involved in the three components of our policies (Table 1): pretrial release for non-felons, pretrial release for felons, and split sentences for felons. Each decision entails releasing a defendant or offender for an amount of time, and—in exchange for the increased recidivism risk—we are reducing the jail population by one person for a possibly different amount of time. Our metric, which we call the risk ratio, is the amount of time that a defendant or offender is released divided by the number of jail-days saved, where both of these quantities are conditioned on the person not recidivating. By our modeling assumptions, this ratio is 1.0 for split sentencing because the number of jail-days saved is the same as the number of days on supervision. In contrast, calculating the means of the gamma and lognormal distributions specified in Table 4, we see (Table 6) that the pretrial release of a non-felon achieves an average reduction of 8 jail-days in exchange for a recidivism risk over an average of 128 days (for a ratio of 16) and the pretrial release of a felon achieves an average reduction of 53 jail-days in exchange for a recidivism risk over an average of 191 days (for a ratio of 3.6). Although this ratio is crude—it fails to account for the reduction in jail-days saved due to recidivism on supervision or before case disposition, the larger reduction in jail-days saved if a defendant fails to appear in court, and the future increase in jail population after a recidivist is rearraigned. Nonetheless, this ratio provides a first-order quantification of the nature of these tradeoffs. This ratio reveals that—for a specified risk category (low, medium or high)—split sentencing offers the most favorable tradeoff, followed by the pretrial release for felons, with the pretrial release of non-felons generating the least desirable tradeoff.

**Table 6:** The tradeoff and its ratio. The means in the second and third columns are derived from the gamma and lognormal parameters in Table 4. The risk ratio is the second column divided by the third column. The risk ratio equals 1.0 for split sentencing of felons.

Decision	Mean Increase in Recidivism Exposure (Days)	Mean Reduction in Jail-Days	Risk Ratio
pretrial release of non-felony	128	8	16.0
pretrial release of felony	191	53	3.6

### Main Results of the Numerical Analysis

**Fig 2:**
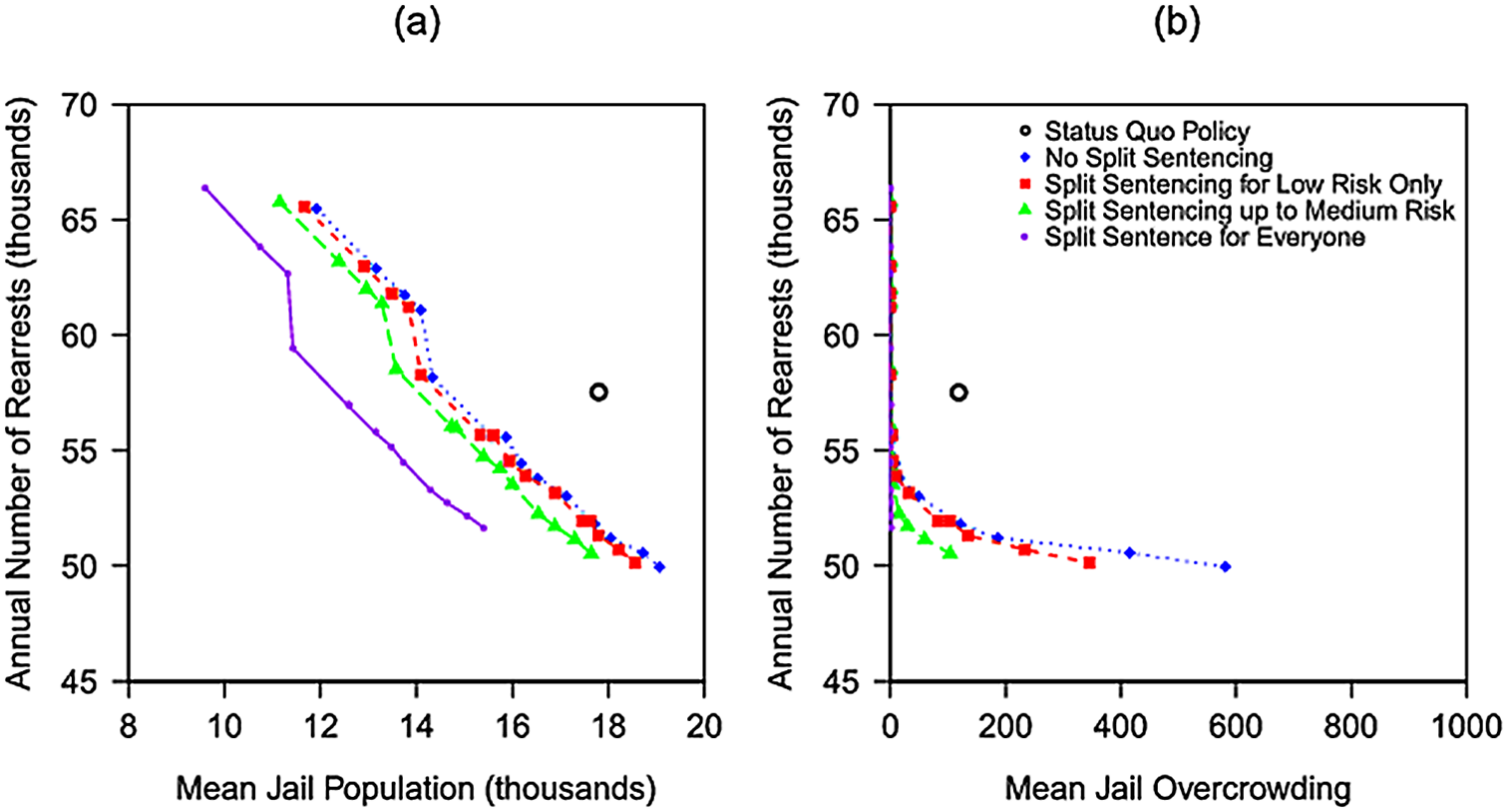
For each of the four options for split sentencing in the right column of Table 1, the optimal (i.e., optimizing over the remaining 16 options in Table 1) tradeoff curves of the annual rearrest rate vs. **(a)** the mean jail population and **(b)** mean jail overcrowding. The circle denotes the status quo policy for LA County in early 2014.

**Fig 3:**
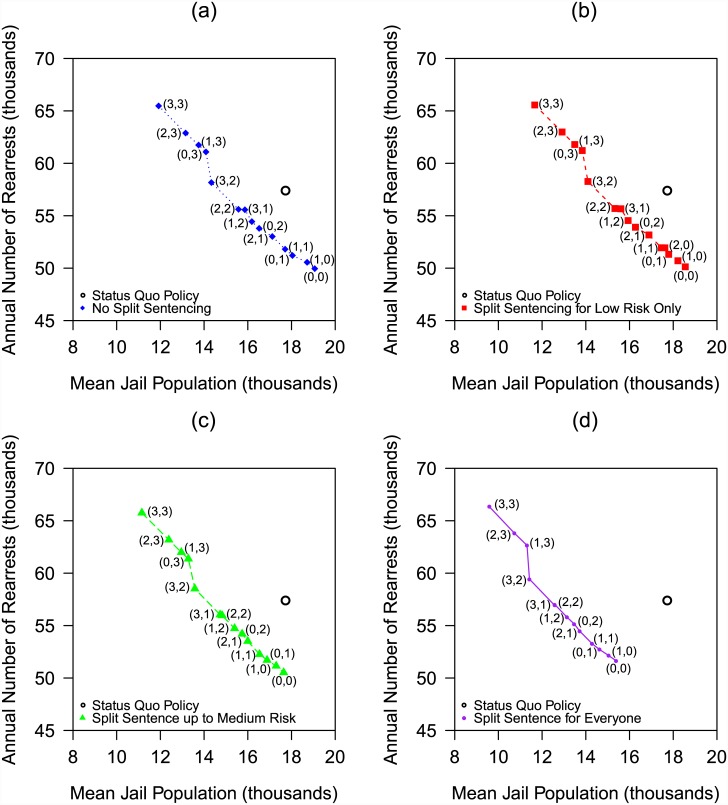
For each of the four tradeoff curves in Fig 2a, the optimal policy along the different points on the curve. Each policy is denoted by a pair of numbers, where the first number corresponds to the pretrial release for non-felonies (left column of Table 1) and the second number corresponds to the pretrial release for felonies (middle column of Table 1).

In our numerical results, the mean jail overcrowding is a nondecreasing function of the mean jail population; consequently, we focus most of our discussion on the tradeoff between the annual rearrest rate and the mean jail population. Our main results (Fig 2a) show the performance of the status quo policy and four tradeoff curves, one for each of the split-sentencing options in Table 1. Each of the four tradeoff curves connects up to 16 points, one for each of the 16 possible pretrial release policies in Table 1. The optimal pretrial release policies for these four tradeoff curves are specified in Fig 3, using the numbering system in Table 1. In total, eight of the 64 policies are dominated by other policies that use the same split-sentencing option (i.e., these other policies achieve simultaneous reductions in rearrest rate and mean jail population) and do not appear in Fig 3. Not surprisingly (Table 5), the dominated policies favor the pretrial release of non-felons over felons; e.g., in Fig 3a, the two dominated pretrial release policies are (2,0) and (3,0), using the numbering system in Table 1. For a given split-sentencing option, we connect these points to create a tradeoff curve only for ease of visualization, and did not assess the performance of any policies that randomize between different points on a tradeoff curve.

The main insight from Fig 2a is the importance of offering split sentencing to high-risk felons. The reduction in the performance measures between the tradeoff curve that offers split sentencing to all felons and the tradeoff curve that offers split sentencing to only low- and medium-risk felons is much larger than the reduction in the performance measures between the tradeoff curve offering split sentencing to only low- and medium-risk felons and the tradeoff curve offering no split sentencing. This result is due to the low risk ratio of split sentencing (Table 6), coupled with the facts that convicted felons make up a significant portion of the jail population (Table 5) and the majority of them are high risk (Table 4). Relative to the status quo policy, the tradeoff curve that provides split sentencing to all felons achieves a 29% reduction in the jail population level at the same rearrest rate, or, e.g., simultaneously reduces the jail population by 20% and the rearrest rate by 7%. The suboptimality of the status quo policy relative to all four of these tradeoff curves stems from the fact that the status quo policy is not purely risk-based (Table 2).

The four curves in Fig 2a do not exhibit the strong convexities that would be associated with increasing marginal risk at a higher release rate. The one exception is the near vertical jump from (3,2) to (0,3) (Fig 3), where a switch from pretrial releasing all non-felons and some felons to pretrial releasing all felons and no non-felons leads to a very small reduction in jail population but a significant increase in the rearrest rate.

The optimal tradeoff curve among all 64 policies is the lower-left envelope of the four tradeoff curves, which consists of the entire leftmost (i.e., split sentencing for everyone) curve and the bottom portions of the other three curves. The only scenario in which offering split sentencing to all felons is not optimal is where it is deemed important to minimize the total rearrest rate below 51.5k/yr (which is the lowest level achievable by any policy that offers split sentencing to all felons). Comparing the lower endpoints of the two leftmost curves in Fig 2a, we see that disallowing split sentencing for high-risk felons while continuing to offer split sentencing to other felons and pretrial release to everyone, the average annual rearrest rate can be decreased by 2%, but the jail population increases by 14%. This change represents a much less attractive tradeoff than when the total arrest rate is > 51.5k/yr; i.e., the slope of the leftmost curve in Fig 2a is much steeper than the slope of the line segment (not pictured in Fig 2a) that connects the lower endpoints of the two leftmost curves. In contrast, if the primary concern is with jail overcrowding rather than the mean jail population, then less aggressive jail population reduction policies can be considered because many of the policies from Table 1 totally eliminate jail overcrowding (Fig 2b; the analog of Fig 3 for the jail overcrowding metric appears in Fig C in S1 File).

Despite the risk ratios in Table 6, it may not be practical to use a more aggressive pretrial release policy for felonies than for non-felonies. Consequently, we restrict our consideration of policies to those that treat felonies at least as strictly (with respect to pretrial release) as non-felonies of the same risk category; i.e., we only allow pretrial release policies (i,j) such that i≥j. The resulting tradeoff curves with this additional restriction appear in Fig D in S1 File, and are nearly linear.

## Discussion

While LA County may be the most prominent example, many other counties in CA [1, 2] and throughout the U.S. are struggling with overcrowding. Our model allows us to assess how pretrial release and split-sentencing decisions impact the key tradeoff between public safety and jail congestion. Our approach—assessing the incarcerated population vs. public safety tradeoff in a queueing network where flows are dictated by risk-based models—should be applicable for addressing a range of policies (e.g., related to drug offenses) in other criminal justice systems (e.g., Federal prisons). In our view, the study’s main contributions are in (i) developing a mathematical model that captures the salient features of the problem and provides a framework for quantifying the tradeoff between public safety and jail congestion, (ii) introducing a simple metric—the risk ratio in Table 6—that sheds light on the varying amount of risk inherent in each type of decision in Table 1, (iii) identifying key data needs, and (iv) highlighting key assumptions and issues. However, there are a number of limitations to our study that need to be addressed before our main results can be directly applied. Hence, before discussing our main results, we describe the limitations of the study, from a data and a structural viewpoint.

### Limitations

There are several shortcomings in the data. First, the survival model used to determine the time to recidivism is the same, regardless of whether a person is out on pretrial release, has failed to appear in court, is on probation after a non-felony, or is on supervision during the latter part of a split sentence for a low-level felony. Moreover, the recidivism model is calibrated using pre-alignment state parolee data, which is a different population than the post-alignment jail population that is the focus of our model. Because many state prisoners and parolees became the responsibility of the county jail system during realignment, it is possible that the pre-alignment parole population does not behave very differently than the post-alignment jail population. But before our model can be applied to a CA county jail system, the risk models commonly used in CA (e.g., CSRA and COMPAS) need to be validated separately for the jail subpopulations that are on pretrial release, after failure to appear in court, non-felony probation and low-level felony supervision.

On a similar note, we also estimate the risk profile of defendants entering arraignment in our model from the risk profile of released state parolees during pre-alignment. Because many offenders arraigned on non-felony charges have a felony background, this assumption may not be as bold as it appears at first blush. Nonetheless, our model needs to use the risk profile of the actual jail population before it can be reliably used. We also assume that risk category and charge type are statistically independent, and this assumption should be investigated, which requires separate risk distributions for non-felons and felons.

The only failure-to-appear data we found that is sufficiently detailed for our purposes are from Broward County, FL [17]. The data in [17] do not include the time of each defendant’s case disposition, which prevents us from assessing (e.g., via logistic regression) whether the likelihood of appearing in court is impacted by the arraignment-to-disposition delay. More importantly, Broward County may have a different defendant population than LA County. The failure-to-appear probability in [17] is consistent with other estimates from KY (page 2 in [26]) and a nationwide study of federal prisoners [22], although much lower than a 0.4 estimate from a large urban center [27]. As a sensitivity analysis, we recompute the tradeoff curves in Fig 2, but change the failure-to-appear probabilities to 0.279 for low risk and 0.425 for medium and high risks, so that the mean failure to appear is 0.4 (i.e., 0.170(0.279)+0.83(0.425) = 0.4) and the risk ratio of the probabilities is the same as in the base case values in Table 3 (i.e., 0.117/0.178 = 0.279/0.425). As expected, these new tradeoff curves (Fig E in S1 File) favor split sentencing over pretrial release even more than in Fig 2. Hence, our use of failure-to-appear data in [17] is a conservative decision with respect to our main qualitative conclusion.

The CA jail system continues to be in flux due to the November 4, 2014 passage of Proposition 47 (Safe Neighborhoods and Schools Act), which reclassifies several drug- and theft-related offenses as non-felonies and allows for resentencing of previously convicted felons. This change altered the composition and total population of CA jails, and the immediate reduction in jail population in November 2014 was largely counteracted by an increase in time served for traditional jail inmates (pages 3–4 in [5]). Our model needs to account for these recent changes before it can be used in CA.

A provocative aspect of our model—and indeed of the U.S. correctional system—is that the time from arraignment to case disposition, the court outcomes and the length of post-sentencing jail terms depend on whether the offender is released or held in custody prior to trial. More specifically, the delays are shorter, the outcomes are more severe and the jail terms are longer for those held in custody. These phenomena have been observed elsewhere in the literature (§2b of [28]), even after controlling for prior convictions, the severity of the current charge, and the strength of the evidence against the defendant [29]. However, we assume that these structural differences, which can be seen by comparing the columns in Table 4, hold regardless of the aggressiveness of the pretrial release policy (Table 1). As a result, awarding a defendant pretrial release reduces the jail population in our model in three ways: it keeps the offender out of jail before case disposition, it reduces the likelihood that he is returned to jail at case disposition, and it reduces his jail time if he is returned to jail. There may be some merit in this assumption (beyond the results in [28, 29]) because those on pretrial release have a greater opportunity to impress jurors and judges (e.g., by appearing in a socially acceptable attire instead of a jail uniform, maintaining a job and not recidivating) and to provide a strong legal defense [28]. However, it is also likely that the pretrial release decisions are based partially on data that are not included in our model; e.g., that judges set higher bonds—leading to less likely pretrial release—when they view the probability of acquittal as low [30], or when they incorporate retribution concerns (e.g., LA County does not accept inmates with bail < $25k for non-felonies, and so courts set the bail ≥ $25k if they want to guarantee detention [6]). To the extent that this is true, we may be overestimating the benefit (in terms of a reduction in jail population and retribution) of pretrial release. However, because our main result is that an aggressive split-sentencing policy is optimal, this assumption—by overstating the benefits of pretrial release—is conservative.

Our mathematical model includes six types of time measurements that are fit to probability distributions: interarrival times to the jail, time delay from arrest to arraignment, time to recidivism, time from arraignment to disposition, post-sentence jail term, and length of probation. In each case, we do not have data for an empirical distribution that would guide the specific choice of probability distribution (the closest we have is a probability mass function with seven time buckets for post-sentence jail terms, see Fig B in S1 File). Instead, we have more aggregate data and choose a commonly-used two-parameter distribution (or compare two of these distributions) such as lognormal, gamma and Erlang. Although there is precedent for using several of these distributions in practice (e.g., the split lognormal model has been used in previous recidivisim studies [20] and the Erlang distribution is widely used to model interarrival times that have coefficient of variation less than one [31], the model’s reliability would be strengthened if there was more evidence that the specified probability distributions were close to the corresponding empirical distributions.

Another implicit set of assumptions is the model’s boundaries. While the correctional system has many interacting parts [2], we consider several important aspects as exogenous. One aspect is court processing capacity and prosecution behavior, both of which can delay case disposition [28]. Note that by assuming exogenous delays until case disposition, we do not capture the counterintuitive fact (implied by priority queueing theory in equation (3.42) in [31] under the assumptions of a Markovian system with nonpreemptive priority) that if there is an increase in the pretrial release rate, the average waiting times (i.e., the time from arraignment to case disposition) for those in pretrial custody and those in pretrial release both decrease (even though the mean overall waiting time remains the same). Another exogenous aspect of the model is probation capacity and related rehabilitation services capacity. Indeed, the stated goal of CA realignment is to reduce recidivism of low-level felons by localizing their rehabilitation services [2]. Although the number of probationers increased during realignment, LA County has greatly enhanced staffing and has reached most of its goals for caseload per probation officer (Table 3 in [32]). We also take policing capacity and crime clearance policy as exogenously specified. Finally, we note the possibility that risk models such as CSRA and COMPAS—by including a defendant’s criminal history to predict the level of risk—may be reinforcing the cumulative disadvantage in sentencing experienced by Black defendants [33].

Our results naïvely assume that all of these policies are fully enforceable. In practice, even though the proportion of eligible low-level felons receiving split sentences increased from < 1% in May 2014 to 27.7% in Feb 2015 (Fig 6 of [5]), many judges were not offering split sentences in the months after the passage of AB 1468. Moreover, some low-level felons may prefer to spend their entire sentence in jail rather than on mandatory community supervision (pg 12 in [34]). Similarly, the predicted improvements achieved by pretrial release will inevitably require a shift from a cash-based bail process to a risk-based bail process [28, 35]. Finally, we note that for simplicity and for equity concerns, we consider only policies that are independent of the size of the current jail population. In other queueing systems with no waiting rooms, such as some telecommunications systems, it is known that system performance can be enhanced by using flow management policies that depend on the current queue length [36].

### Main Results

Our model predicts that offering all low-level felons—including those in the high-risk category—split sentences is the key to achieving a substantial improvement in performance, and can simultaneously reduce the mean jail population level and the annual rearrest rate relative to the status quo policy that attempts to mimic LA County’s policy in early 2014. This result is not inconsistent with the observation in the prison setting that the most effective way to substantially reduce the prison population is to focus on prisoners who serve long sentences [37].

In addition, we introduce the risk ratio metric (Table 6), which explains why—for a given CSRA risk level—split sentencing for felonies is more effective than pretrial release for felonies, which in turn is more effective than pretrial release for non-felonies. The large risk ratios for pretrial release are due to the courts prioritizing cases of defendants in custody over cases of defendants under pretrial release; indeed, the pretrial risk ratios would be 1.0 in Table 6—the same as the risk ratio for split sentencing—if the courts processed cases in a first-come first-served manner. In light of the fact that prioritizing defendants in custody reduces the jail population relative to using first-come first-served, the high risk ratios in Table 6 are due to a systemic aspect of the criminial justice system that is not easily fixed (e.g., without significantly increasing court capacity), and hence split sentencing is intrinsically a more attractive option than pretrial release from a purely operational standpoint.

The slopes of the curves in Fig 2 are ≈ −2 crimes per jail-year, which is somewhat higher than the empirical estimate of −1.2 crimes per prison-year from CA realignment (although their estimate from a simpler cross county model is −2.5 crimes per prison-year) [38]. It would be interesting to understand the reason for this discrepancy, particularly whether it is due to improved supervision or the localization of rehabilitation services during realignment. The additional crimes due to prison downsizing in [38] were not violent and were dominated by auto thefts, at a cost of $9533 per crime. The differential cost of detention relative to supervision is ≈ $40k/yr ($113/day for detention [39] minus $1533/yr for probation, page 12 in [40], implying a marginal return from incarceration of ≈ 50%, which is higher than the marginal return of 23% in [38]. In either case, a comparison with the marginal returns from additional police of 160% [41] or additional substance abuse disorder treatment of 156–300% [42] suggests that there may be more cost-effective ways than incarceration to reduce crime. The decision of where to reside on the lower left envelope of the tradeoff curves in Fig 2 can be reduced in our model to a tradeoff of the reduced detention cost (at $113/day [39]) and the increased crime cost (e.g., from [38]), but this type of analysis is only one input into a very complex societal issue.

### Conclusion

Although our results need to be confirmed by calibrating every aspect of the model with data from a single county, they suggest that split sentencing for all low-level felons is the key lever in managing the tradeoff between public safety and jail congestion, as demonstrated by a representative model and a powerful yet simple metric.

## Supporting Information

S1 File Supporting Material. Contains the statistical estimation of model parameters and provides tradeoff curves for secondary results.
(PDF)

## References

[1] California State Department of Finance. AB 1468 Report. Sacramento, CA 1 15, 2015.

[2] Petersilia J. California prison downsizing and its impact on local criminal justice systems. *Harvard Law & Policy Review* 8, 327–357, 2014.

[3] Yang M., Wong S. C. P., Coid J. The efficacy of violence prediction: a meta-analytic comparison of nine risk assessment tools. *Psychological Bulletin* 136, 740–767, 2010 10.1037/a0020473 20804235

[4] Turner S., Hess J., Jannetta J. Development of the California Static Risk Assessment Instrument (CSRA) Center for Evidence-Based Corrections working paper, UC Irvine, Irvine, CA, 11 2009.

[5] Powers, J. E., Delgado, M. Public safety realignment implementation—May 2015 update. Countywide Criminal Justice Coordination Committee, May 5, 2015.

[6] Vera Institute of Justice. Los Angeles County jail overcrowding reduction project, final report: revised. Vera Institute of Justice, New York, NY, 9 2011.

[7] Freedman A. E., Lynn-Whaley J., Carmody K., Rosenbaum B. Santa Clara County AB109 public safety realignment interim evaluation. Resource Development Associates, 3 6, 2013.

[8] Sharkey J., Cosden M., Hunnicutt K., Donahue M., Schiedel C. Public safety realignment in Santa Barbara County, Preliminary evaluation report, UCSB, Santa Barbara, CA, 2013.

[9] VanNostrand M., Keebler G. Pretrial risk assessment in the federal court. U.S. Dept. of Justice, Washington, D.C., 4 14, 2009.

[10] Hickert A., Worwood E. B., Prince K. Pretrial release risk study, validation, & scoring: final report Utah Criminal Justice Center, U. of Utah, Salt Lake City, UT, 4 2013.

[11] Austin, J., Naro-Ware, W., Ocker, R., Harris, R., Allen, R. Evaluation of the current and future Los Angeles County jail population. JFA Institute Report, April 10, 2012.

[12] American Civil Liberties Union of Southern California. Los Angeles County Jail plan introduced 2/6/13. Memo, February 19, 2013.

[13] LA County Sheriff’s Department Year in review 2012. Monterey Park, CA, 2013.

[14] CA Department of Corrections and Rehabilitation. COMPAS assessment tool launched. Accessed at http://www.cdcr.ca.gov/rehabilitation/docs/FS_COMPAS_Final_4-15-09.pdf on June 10, 2015.

[15] Beard J., Toche D., Beyer B., Babby W., Allen D., Grassel K., *et al* 2013 outcome evaluation report. California Department of Corrections and Rehabilitation, 1 2014.

[16] Farabee D., Zhang S., Roberts R. E. L., Yang J. COMPAS validation study: final report Semel Institute for Neuroscience and Human Behavior, UCLA, Los Angeles, CA, 8 15, 2010.

[17] Blomberg T., Bales W., Mann K., Meldrum R., Nedelec J. Validation of the COMPAS risk assessment classification instrument College of Criminology and Criminal Justice, Florida State University, Tallahassee, FL, 9 2010.

[18] Schmidt P., Witte A. D. Predicting criminal recidivism using “split population” survival time models. *J. Econometrics* 40, 141–160, 1989 10.1016/0304-4076(89)90034-1

[19] Kalbfleisch J. D., Prentice R. L. *The statistical analysis of failure time data*. John Wiley & Sons, New York, NY, 1980.

[20] Chung C.-F., Schmidt P., Witte A. D. Survival analysis: a survey. *J. Quantitative Criminology* 7, 59–98, 1991 10.1007/BF01083132

[21] Jahr S. Court realignment data—calendar year 2013. Judicial Council of CA, San Francisco, CA, 8 22, 2014.

[22] Lowenkamp C. T., Lemke R., Latessa E. The development and validation of a pretrial screening tool. *Federal Probation* 72, 3, 2–9, 2008.

[23] Delgado, M. Public safety realignment implementation update—year one report. Countywide Criminal Justice Coordination Committee, November 28, 2012.

[24] Shouse California Law Group. Misdemeanor (summary) probation in California. Accessed at www.shouselaw.com/misdemeanor-probation.html on June 19, 2015.

[25] Shouse California Law Group. How “felony probation” works in California. Accessed at www.shouselaw.com/felony-probation.html on June 19, 2015.

[26] Cate J. Written testimony for March 21, 2013 hearing of Little Hoover Commission, CA State Association of Counties, Sacramento, CA, 2013.

[27] Berk R., Bleich J., Kapelner A., Henderson J., Barnes G., Kurtz E. Using regression kernels to forecast a failure to appear in court Dept. of Criminology, U. Pennsylvania, Philadelphia, PA, Auguest 23, 2014.

[28] Manns J. Liberty takings: a framework for compensating pretrial detainees John M. Olin Center for Law, Economics, and Business, discussion paper no. 512, Harvard University, Cambridge, MA, 2005.

[29] Frazier C. E., Bishop D. M. The pretrial detention of juveniles and its impact in case dispositions. *J. Criminal Law & Criminology* 1132, 1139–1152, 1985.

[30] Landes W. M. Legality and reality: some evidence on criminal procedure. *J. Legal Studies* 287, 333–335, 1974.

[31] Gross D., Harris C. M. *Fundamentals of queueing theory*, 2nd edition John Wiley & Sons, New York, 1985.

[32] Public Safety Realignment Team. Public safety realignment: year-three report. County of Los Angeles, CA, 1 2015.

[33] Wooldredge J., Frank J., Goulette N., Travis L. III Is the impact of cumulative disadvantage on sentencing greater for Black defendants? *Criminology & Public Policy* 14, 187–223, 2015 10.1111/1745-9133.12124

[34] Barton-Bellessa S. M., Hanser R. D.. Community-based corrections: a text/reader. Sage Publications, Thousand Oaks, CA, 2012.

[35] Pretrial Justice Institute. Rational and transparent bail decision making: moving from a cash-based to a risk-based process. Gaithersburg, MD, 3 2012.

[36] Kelly F. P. Loss Networks. *Annals Applied Probability* 1, 319–378, 1991 10.1214/aoap/1177005872

[37] Tonry M. Remodeling American sentencing: a ten-step blueprint for moving past incarceration. *Criminology & Public Policy* 13, 503–533, 2014 10.1111/1745-9133.12097

[38] Lofstrom M., Raphael S. Incarceration and crime: evidence from California’s realignment sentencing reform Goldman School of Public Policy, U. California, Berkeley, CA, 2015.

[39] Greene, J. A. The cost of responding to immigration detainers in California: preliminary findings. Justice Strategies, August 22, 2012.

[40] Golden M., Siegel J., Forsythe D. Cost-benefit analysis. Vera Institute of Justice, New York, NY 2006.

[41] Chalfin A., McCrary J. Are U.S. cities underpoliced?: theory and evidence Berkeley Law School, U. California, Berkeley, CA, 2013.

[42] Wen, H., Hockenberry, J. M., Cummings, J. R. The effect of substance abuse disorder treatment use on crime: evidence from public insurance expansions and health insurance parity mandates. National Bureau of Economics Research paper no. 20537, 2014.

[43] Board of State and Community Corrections California. About the jail population dashboard. Accessed at https://public.tableau.com/profile/kstevens#!/vizhome/ACJR-October2013/About on July 19, 2015.

[44] Delgado, M. Public safety realignment implementation update—December 2012 to January 2013. Countywide Criminal Justice Coordination Committee, March 4, 2013.

[45] Public Safety Realignment Team. Public safety realignment: year-two report. County of Los Angeles, CA, 12 2013.

