# Supplementary material for: Assessing Risk-Based Policies for Pretrial Release and Split Sentencing in Los Angeles County Jails
Source: PLoS One. 2015 Dec 29;10(12):e0144967. doi: 10.1371/journal.pone.0144967 (PMC4694647; doi:10.1371/journal.pone.0144967)
Supplement: S1 File — Contains the statistical estimation of model parameters and provides tradeoff curves for secondary results. (PDF) [file pone.0144967.s001.pdf]

SUPPORTING MATERIAL

A Status Quo Pretrial Release Probabilities

To derive the six probabilities of pretrial release conditioned on risk category and charge that appear in Table 2 in the main text, we proceed in three steps: compute the pretrial detention probability conditioned only on risk category, then compute the pretrial detention probability conditioned only on charge, and then derive the probability of pretrial detention conditioned jointly on risk and charge.

We first derive the probability that a defendant of each risk category (regardless of charge) is released prior to trial, using a nationwide study of federal courts [1] and a study of Salt Lake County (UT) jails [2]. We note that – like LA County – the federal courts and Salt Lake County allow for commercial bail, bounty hunters and a uniform bail schedule. On page 31 of [1], risk categories 1 and 2 correspond to low risk, category 3 corresponds to medium risk, and categories 4 and 5 correspond to high risk. The five risk categories have equal numbers of defendants in them. By Fig. 23 in [1], the release probabilities are (0.871+0.623)/2=0.747 for low risk, 0.494 for high risk, and (0.400+0.279)/2=0.3395 for high risk.

The seven risk categories in [2] are equally probable by construction, with risk categories 1, 2-3, and 4-7 corresponding to low, medium and high risk (Table 17 in [2]). We denote the events that a defendant belongs to the low, medium and high risk category by L, M and H, and denote the events of pretrial release and detention by R and D. Our goal is to compute conditional probabilities $P(\mathrm{R}|\mathrm{L})$, $P(\mathrm{R}|\mathrm{M})$ and $P(\mathrm{R}|\mathrm{H})$. By construction of the risk categories, we know P(L)=0.143, P(M)=0.286 and P(H)=0.571. Table 19 of [2] gives the conditional probabilities $P(\mathrm{L}|\mathrm{R})$=0.43, $P(\mathrm{L}|\mathrm{D})$=0.25, $P(\mathrm{M}|\mathrm{R})$=0.26, $P(\mathrm{M}|\mathrm{D})$=0.22, $P(\mathrm{H}|\mathrm{R})$=0.31 and $P(\mathrm{H}|\mathrm{D})$=0.53. On page 5 of [2], the sample size includes 4986 defendants with pretrial release interviews and 2981 without pretrial release interviews. Of a random sample of 1456 from

the 4986 defendants with pretrial release interviews, 390 were not released. Assuming that all 2981 defendants without pretrial release interviews were detained, we estimate that (1-390/1456)4986= 3650 were released and (390/1456)4986+2981 = 4317 were detained, giving P(R)=0.458 and P(D)=0.542. We substitute all of these probabilities into the law of total probability to get

$$P(\mathrm{R}|\mathrm{L}) = \frac{P(\mathrm{L}|\mathrm{R})P(\mathrm{R})}{P(\mathrm{L}|\mathrm{R})P(\mathrm{R}) + P(\mathrm{L}|\mathrm{D})P(\mathrm{D})} = 0.593. \tag{A}$$

Replacing L in (A) by M and H yields P(R|M)=0.500 and P(R|H)=0.331. Finally, taking an average of these conditional probabilities over the two studies [1, 2], we assume that the probability of release for a defendant of low, medium or high risk is 0.667, 0.500 and 0.333, respectively. Therefore, the conditional probabilities of pretrial detention given risk category are P(D|L)=0.333, P(D|M)=0.500 and P(D|H)=0.667.

Having derived the pretrial detention probabilities conditioned on risk, we now compute the pretrial detention probabilities conditioned on charge. By the top right box on page 79 of [3], the probability that a felon in LA County was released is 0.23 and the probability that a defendant with a non-felony in LA County was released is 0.66. Letting N denote non-felony and F denote felony, the conditional probabilities of pretrial detention given charge are P(D|N)=0.34 and P(D|F)=0.77. Using the risk proportions in Table 1 of the main text, we find that the probability of being detained is P(D)=0.549(0.667)+0.281(0.500)+0.170(0.333) =0.563.

Our goal now is to derive a mathematical expression for the conditional probability of pretrial detention given the charge type C (either N or F) and risk category R (either L, M or H), which we denote by P(D|C,R), in terms of the probabilities P(D|R), P(D|C) and P(D) calculated above. By our assumption that C and R are independent, it follows that P(C|R)=P(C) and P(C|R,D)=P(C|D). By the definition of conditional probability, we have P(C|D)=P(C,D)/P(D) and P(C|R,D)=P(C,D|R)/P(D|R). Substituting the right sides

of these two equations into P(C|R,D)=P(C|D) yields P(C,D|R)/P(D|R)=P(C,D)/P(D). Substituting P(D|C)P(C) for P(C,D) in this equation and rearranging gives

$$\frac{P(\mathrm{C},\mathrm{D}|\mathrm{R})}{P(\mathrm{C})}=\frac{P(\mathrm{D}|\mathrm{C})P(\mathrm{D}|\mathrm{R})}{P(\mathrm{D})}. \tag{B}$$

Finally, we find that

$$P(\mathrm{D}|\mathrm{C},\mathrm{R}) = \frac{P(\mathrm{D},\mathrm{C}|\mathrm{R})}{P(\mathrm{C}|\mathrm{R})}, \tag{C}$$

$$= \frac{P(\mathrm{D},\mathrm{C}|\mathrm{R})}{P(\mathrm{C})} \text{ because C and R are independent,} \tag{D}$$

$$= \frac{P(\mathrm{D}|\mathrm{C})P(\mathrm{D}|\mathrm{R})}{P(\mathrm{D})} \text{ by } (B). \tag{E}$$

Substituting the numerical values above into the right side of (E) and then noting that the conditional probability of release given crime type and risk category is 1-P(D|C,R), we get the conditional release probabilities in Table 2 of the main text.

B Pretrial Release After Recidivism

According to Table 2 of [3], 20-21% of felons in LA County receive financial release. Table 19 in the Appendix of [4] states that 19% of felons receive financial release in LA County, confirming the numbers in [3]. When averaged over the 75 largest counties in the U.S., Table 6 in [4] states that 33% of felons receive financial release and Table 8 in [4] states that 18% of felons with custody history receive financial release. Extrapolating these nationwide numbers back to LA County, we estimate that 18(19)/33=10.4% of felons in LA County with custody history receive financial release. For lack of data on non-felony defendants, we assume that 20% (10%, respectively) of defendants charged with non-felonies (respectively, felonies) during a recidivism event receive financial release.

C Interarrival Times

The arraignments under custody from the LA County Sheriff's Department's Year in Review reports (e.g., [5]) for the years 2008-2012 give an average of 350.4/day with no obvious long-term trend (Table A). There is little variability over the time of year (page 36 of Appendix C in [3]), and we ignore the fact that fewer arraignments occur over the weekend (page 34 of Appendix C in [3]). We estimate the squared coefficient of variation (i.e., the variance divided by the square of the mean) of the interarrival times to be 0.465 from the data in Table A by assuming that the times between consecutive arrivals are independent and identically distributed. The Erlang distribution is characterized by its shape parameter and scale parameter. The Erlang shape parameter is the reciprocal of the squared coefficient of variation, and so we set the shape parameter equal to two. The mean of an Erlang distribution is the product of its shape and scale parameters. Because the arrival rate of 350/day includes recidivists, who account for slightly less than half of the total number of arrivals, we adjust the rate of new arrivals to be 193/day so that the total arrival rate is 350/day. This yields a shape parameter of 1/395.

D Time to Arraignment

Arraignment data from LA County in 2008 states that 4% of defendants were arraigned within 24 hours of arrest, 56.4% were arraigned within 48 hours, 70.4% were arraigned within 72 hours, and 95% were arraigned within 96 hours (page 63 of [3]). The maximum likelihood estimate for the lognormal parameters from these data yield the values in Table 3 of the main text.

E Time to Recidivism

Let the cumulative distribution function (CDF) $F(t;\Theta,j)$ be the probability that an offender recidivates within t time units of being released, given that he is of risk category j (where $j=1,2,3$ correspond to low, medium and high risk) under parameter set Θ. These CDFs are specified for our five models in Table B. Let N_j be the number of offenders in the CSRA cohort [6] with risk category j, where $N=\sum_{j=1}^{3} N_j$. Let N_{jk} be the number of offenders in the CSRA cohort with risk category j that did not recidivate within $k-1$ years but did recidivate within k years. The data from [6] are $N=110,313$, $N_1=18,768$, $N_2=31,024$, $N_3=60,521$, $N_{11}=4845$, $N_{12}=1681$, $N_{13}=641$, $N_{21}=12,002$, $N_{22}=3403$, $N_{23}=1269$, $N_{31}=33,243$, $N_{32}=7925$ and $N_{33}=2584$. We choose the values of Θ to maximize the log-likelihood function,

$$\sum_{j=1}^{3} N_{j1}\log F(1;\Theta,j)+\sum_{j=1}^{3} N_{j2}\log(F(2;\Theta,j)-F(1;\Theta,j))$$
$$+\sum_{j=1}^{3} N_{j3}\log(F(3;\Theta,j)-F(2;\Theta,j))+\sum_{j=1}^{3}(N_j-N_{j1}-N_{j2}-N_{j3})\log(1-F(3;\Theta,j)). \quad \text{(F)}$$

The estimation results (Table C) suggest that the lognormal models outperform the proportional hazards models (as measured by the negative log-likelihood values), and the split is statistically significant in the lognormal model only if we incorporate risk-associated heteroskedasticity. We use the split lognormal model with heteroskedasticity, and the three probability density functions (one for each risk category) are pictured in Fig. A. Using quarterly average cumulative recidivism probabilities for three years from Fig. 2 of [6], we perform a granular cross-validation and find that the pointwise root mean square error between the predicted (by the split lognormal model with heteroskedaticity) and actual probabilities is 0.022, with a pointwise maximum difference of 0.049.

F Failure To Appear

We use a validation study of COMPAS for predicting failure to appear in court in Broward County, FL [7]. COMPAS's 10 risk scores were aggregated into CSRA's three risk categories, where scores 1-4, 5-7 and 8-10 correspond to low, medium and high risk (page 20 of [7]). Table 13 in [7] gives failure-to-appear probabilities for the three risk groups at six different follow-up periods, but does not provide information on the time from arraignment to disposition. Hence, we cannot attempt to estimate (e.g., via logistic regression) whether the failure-to-appear probability depends on the arraignment-to-disposition delay. To minimize right-censoring (i.e., due to a case disposition date that is far in the future), we use their largest follow-up period of 12 months; these data are reproduced in Table D. Because the failure-to-appear probability is slightly higher for medium risk than high risk (the same is true for follow-up periods of three months and six months in Table 13 in [7]), we combine medium risk and high risk in our analysis. Hence, we assume that the failure-to-appear probability is 0.117 for low risk and is [456(0.180)+163(0.172)]/(456+163)=0.178 for both medium risk and high risk.

G Time from Arraignment to Case Disposition

Because the time from arrest to arraignment is typically much smaller than the time from arraignment to disposition, we use arrest-to-disposition time data from pages 55-56 in [3], which appear in Table E, to estimate the time from arraignment to disposition, using both gamma and lognormal distributions. Lognormal provides a better fit (using the Kolmogorov distances in Table E) for felonies in pretrial release, and gamma (with an increasing failure rate for those released and a decreasing failure rate for those in custody) provides a better fit for felonies under pretrial custody and for all non-felonies.

H Case Disposition Probabilities

The 14 case disposition probabilities in Table 4 in the main text are derived in three groups. Ignoring the ongoing cases in Table 4 of [3] and combining acquitted and dismissed cases, we estimate the four proportion dismissed values that appear in Table 4 in the main text (e.g., for felons on pretrial release, (1635+46)/(12,154-1168)=0.153).

We denote the proportion of non-felony charges that are put on probation after pretrial release, put on probation after pretrial custody, receive a jail sentence after pretrial release and receive a jail sentence after pretrial custody by p_1, p_2, p_3 and p_4, respectively. We jointly solve for these four unknowns using four equations (K)-(P). The first two equations use the proportion dismissed values, 0.207 and 0.052, in Table 4 in the main text:

$$p_1 + p_3 = 1 - 0.207, \tag{G}$$

$$p_2 + p_4 = 1 - 0.052. \tag{H}$$

The following equation specifies that the proportion of all non-felony charges that result in jail sentences is 7310/16,891 = 0.433 (page 129 of [3]):

$$0.66p_3 + (1 - 0.66)p_4 = 0.433, \tag{I}$$

where 0.66 was estimated in §A. The following equation uses the result that the odds ratio to be jailed for a non-felony in pretrial custody relative to a non-felony under pretrial release is 4.44 (page 10 in [8]):

$$\frac{p_4(1 - p_3)}{p_3(1 - p_4)} = 4.44. \tag{J}$$

Solving (G)-(J) jointly gives the proportions in Table 4 in the main text.

The remaining six case disposition probabilities are derived jointly. We denote the proportion of felons who are put on probation after pretrial release, put on probation after pretrial custody, receive a jail sentence after pretrial release, receive a jail sentence after

pretrial custody, are sent to prison after pretrial release, and are sent to prison after pretrial custody by p_5, p_6, p_7, p_8, p_9 and p_{10}, respectively. We jointly solve for these six unknowns using six equations (K)-(P). The first two equations use the proportion dismissed values, 0.153 and 0.069:

$$p_5 + p_7 + p_9 = 1 - 0.153, \tag{K}$$

$$p_6 + p_8 + p_{10} = 1 - 0.069. \tag{L}$$

The third equation states that the proportion of felons that get probation is, using the last four columns in Table 1 in [9], 23,476/41,725=0.563:

$$0.23p_5 + (1 - 0.23)p_6 = 0.563, \tag{M}$$

where 0.23 was estimated in §A. Similarly, the fourth equation specifies that the proportion of all felons that receive jail sentences is (7407+125)/41,725 = 0.181 (the last four columns in Table 1 in [9]):

$$0.23p_7 + (1 - 0.23)p_8 = 0.181. \tag{N}$$

As in (J), the fifth equation uses the result that the odds ratio to be jailed for a felony in pretrial custody relative to a felony under pretrial release is 3.32 (page 10 in [8]):

$$\frac{(p_8 + p_{10})(1 - p_7 - p_9)}{(p_7 + p_9)(1 - p_8 - p_{10})} = 3.32. \tag{O}$$

The sixth equation states that the probability of pretrial release given a prison charge (as opposed to a prison sentence) is 0.15 (page 57 in [3]), which we derive using Bayes rule as follows. Using the proportion dismissed values 0.153 and 0.069, we find that the probability of a prison charge given pretrial release is $p_9/(1 - 0.153)$, and the probability of a prison charge given pretrial custody is $p_{10}(1 - 0.069)$. Because the probability of pretrial release for

a felony is 0.23 (§A), it follows from Bayes rule that the sixth equation is

$$\frac{0.23\left(\frac{p_9}{1-0.153}\right)}{0.23\left(\frac{p_9}{1-0.153}\right)+(1-0.23)\left(\frac{p_{10}}{1-0.069}\right)}=0.15. \tag{P}$$

Simultaneously solving (K)-(P) gives the proportions in Table 4 in the main text.

I Post-sentence Jail Terms

We fit mixture (of pretrial release and pretrial custody) gamma and lognormal distributions for jail sentences for non-felonies using data on page 129 in [3] and Fig. 25 in Appendix C in [3], and for jail sentences for felonies using data in Chart 3 in [10]. The data (reproduced in Table F) are of the form: N_i offenders have jail terms in the interval $(a_i, a_{i+1}]$ for $i = 1, \ldots, 7$, where $\sum_{i=1}^{7} N_i = N$. We consider the mixture CDF

$$F(x;\Theta)=\frac{w_R}{w_R+w_D}G(x;\Theta_R)+\frac{w_D}{w_R+w_D}G(x;\Theta_D), \tag{Q}$$

where R and D denote the populations that are in pretrial release and pretrial detention, respectively, and w_R and w_D are weights proportional to the sizes of these two populations. Because 66% of non-felonies and 23% of felonies receive pretrial release (§A), and 55.8% of defendants are charged with felonies (Table 3 of the main text), we have that $w_R = 0.66(0.558) = 0.368$ and $w_D = 0.34(0.558) = 0.190$ for non-felonies, and $w_R = 0.23(0.442) = 0.102$ and $w_D = 0.77(0.442) = 0.340$ for felonies. We consider two cases: $G(x)$ is lognormal with parameters (μ, σ) and $G(x)$ is gamma with scale parameter k and shape parameter θ, where R and D are subscripts on the parameters in (Q) to denote the released or detained populations. We use a constrained maximum likelihood approach, where we require the mean jail sentence for non-felonies to be 2.78 times longer for those undergoing pretrial detention relative to those receiving pretrial release, and the mean jail sentence for felonies to be 2.36 times longer for those undergoing pretrial detention relative to those receiving

pretrial release (page 10 of [8]). Hence, setting $r = 2.78$ for non-felonies and $r = 2.36$ for felonies, and setting $F(0;\Theta) = 0$ and $F(\infty;\Theta) = 1$, we solve

$$\min_{\Theta=(\Theta_R,\Theta_D)} \quad -\sum_{i=1}^{7} N_i \ln(F(a_{i+1};\Theta) - F(a_i;\Theta)), \tag{R}$$

$$\text{subject to} \quad e^{\mu_D+\sigma_D^2/2} = re^{\mu_R+\sigma_R^2/2} \ \text{ if } G(x) \text{ is lognormal,} \tag{S}$$

$$\text{subject to} \quad k_D\theta_D = rk_R\theta_R \ \text{ if } G(x) \text{ is gamma.} \tag{T}$$

The results appear in Table G and Fig. B, and we adopt the distribution with the lower negative log-likelihood: gamma for non-felonies and lognormal for felonies.

References

[1] VanNostrand, M., Keebler, G. Pretrial risk assessment in the federal court. U.S. Dept. of Justice, Washington, D.C., April 14, 2009.

[2] Hickert, A., Worwood, E. B., Prince, K. Pretrial release risk study, validation, & scoring: final report. Utah Criminal Justice Center, U. of Utah, Salt Lake City, UT, April 2013.

[3] Vera Institute of Justice. Los Angeles County jail overcrowding reduction project, final report: revised. Vera Institute of Justice, New York, September 2011.

[4] Bureau of Justice Statistics. Felony defendants in large urban counties, 2006. *Bureau of Justice Statistics Bulletin*, NCJ 228944, May 2010.

[5] LA County Sheriff's Department. Year in review 2012. Monterey Park, CA, 2013.

[6] Beard, J., Toche, D., Beyer, B., Babby, W., Allen, D., Grassel, K., Maxwell, D., Nakao, M. 2013 outcome evaluation report. California Department of Corrections and Rehabilitation, January 2014.

[7] Blomberg, T., Bales, W., Mann, K., Meldrum, R., Nedelec, J. Validation of the COMPAS risk assessment classification instrument. College of Criminology and Criminal Justice, Florida State University, Tallahassee, FL, September 2010.

[8] Lowenkamp, C. T., VanNostrand, M., Holsinger, A. Investigating the impact of pretrial detention on sentencing outcomes. Laura and John Arnold Foundation report, Houston, TX, November 2013.

[9] Jahr, S. Court realignment data - calendar year 2013. Judicial Council of CA, San Francisco, CA, August 22, 2014.

[10] Delgado, M. Public safety realignment implementation update - year one report. Countywide Criminal Justice Coordination Committee, November 28, 2012.

Year	Number of Arraignments Under Custody
2008	122,436
2009	126,352
2010	130,959
2011	134,271
2012	125,965

Table A: Raw data to estimate the interarrival time distribution [5].

Model	Parameter Set (Sign Constraint)	CDF
lognormal	$\beta(-)$, μ, $\sigma(+)$	$\frac{1}{2}\left(1+\text{erf}\left(\frac{\ln t-\mu-\beta^T x}{\sqrt{2}\sigma}\right)\right)$
split lognormal	$\beta(-)$, μ,$\sigma(+)$, $\delta(+)$	$\frac{\delta}{2}\left(1+\text{erf}\left(\frac{\ln t-\mu-\beta^T x}{\sqrt{2}\sigma}\right)\right)$
split lognormal with heteroskedasticity	$\beta(-)$, μ,$\sigma(+)$, $\delta(+)$, $\gamma(-)$	$\frac{\delta}{2}\left(1+\text{erf}\left(\frac{\ln t-\mu-\beta^T x}{\sqrt{2}(\sigma+\gamma^T x)}\right)\right)$
proportional hazards	$\beta(+)$, λ	$1-e^{(-\lambda t)^{e^{\beta x}}}$
split proportional hazards	$\beta(+)$, λ, $\delta(+)$	$\delta\left(1-e^{(-\lambda t)^{e^{\beta x}}}\right)$

Table B: Five survival models for time to recidivism.

Model	β	μ	σ	δ	γ	λ	NLL	p-value
lognormal	-1.10<br>-(1.13,1.07)	2.94<br>(2.87,3.01)	2.59<br>(2.55,2.63)				118,698	base
split lognormal	-1.10	2.94	2.59	1.00			118,698	1.00
split lognormal heteroskedasticity	-0.80<br>-(0.84,0.76)	1.74<br>(1.64,1.84)	3.62<br>(3.51,3.74)	0.77<br>(0.76,0.78)	-0.82<br>-(0.78,0.87)		118,310	$< 10^{-3}$
proportional hazards	0.56					0.10	129,390	base
split proportional hazards	0.69			0.69		0.18	120,522	$< 10^{-3}$

Table C: Results from the maximum likelihood estimation in (F), with 95% confidence intervals in parentheses for the two models that provide the best fit. NLL is negative log-likelihood, and p-value is the likelihood ratio test of the enhanced model relative to the base (lognormal or proportional hazards) model.

Risk Category	Sample Size	Failure-to-Appear Probability
low	1901	0.117
medium	456	0.180
high	163	0.172

Table D: Raw data for estimation of the failure-to-appear probabilities, reproduced from Table 13 in [7]. The sample size is the number of defendants in each risk category who did not recidivate during their first 12 months under pretrial release. The failure-to-appear probability is the proportion of these defendants who failed to appear for a court date during their first 12 months of pretrial release.

Charge Type	Pretrial Status	Data (Days)			Gamma			Lognormal		
		Mean	50th	90th	Parameters	K	P	Parameters	K	P
non-felony	release	128.13	91	290	(1.07,119.8)	0.001	0.02	(4.48,0.92)	0.013	6.26*
non-felony	custody	8.23	3	21	(0.46,16.8)	0.016	5.55*	(1.19,1.43)	0.025	14.13***
felony	release	190.83	166	289	(5.27,34.8)	0.031	11.03***	(5.13,0.47)	0.027	7.22**
felony	custody	53.03	29	131	(0.67,76.8)	0.002	0.09	(3.34,1.18)	0.009	3.31

Table E: Fit of gamma and lognormal models to data on time to case disposition. The 50th and 90th columns refer to the median and 90th fractile. The gamma parameters are the shape and scale, the lognormal parameters are the mean and standard deviation of the underlying normal distribution, K refers to the Kolmogorov distance, P refers to the Pearson's goodness-of-fit statistic with three categories (0-29 days, 30-131 days, and > 132 days), and *, ** and *** refer to p-values that are less than 0.05, 0.01 and 0.001, respectively (meaning that there is evidence to reject the null hypothesis that the data are from the specified distribution).

Non-felonies			Felonies		
Interval i	Start of Interval a_i (days)	Population of Interval N_i	Interval i	Start of Interval a_i (months)	Population of Interval N_i
1	0	1827	1	0	4501
2	2	1829	2	8	2001
3	6	440	3	12	1167
4	8	1535	4	18	333
5	31	939	5	24	167
6	61	363	6	30	83
7	91	379	7	36	83

Table F: Raw data for estimation of the length of jail sentences. N_i is the number of offenders whose jail sentence was in the interval $[a_i, a_{i+1})$. We have $a_8 = \infty$ for non-felonies (page 129 in [3]) and $a_8 = 252$ (Chart 3 in [10]). The population values for felonies were derived by multiplying the probabilities in [3] and [10] times the respective sample sizes of 7310 and 8336, and then rounding to the nearest integer.

Charge Type	Pretrial Status	Time Units	Gamma			Lognormal		
			k	θ	NLL	μ	σ	NLL
non-felony	release	days	0.368	29.92	13,030	2.558	1.308	13,082
non-felony	custody	days	0.397	77.08		0.313	2.871	
felony	release	months	1.537	2.550	10,522	1.203	0.633	10,442
felony	custody	months	1.934	4.785		2.064	0.628	

Table G: Results of maximum likelihood estimation in (R)-(T) for lengths of jail sentences. NLL denotes negative log-likelihood.

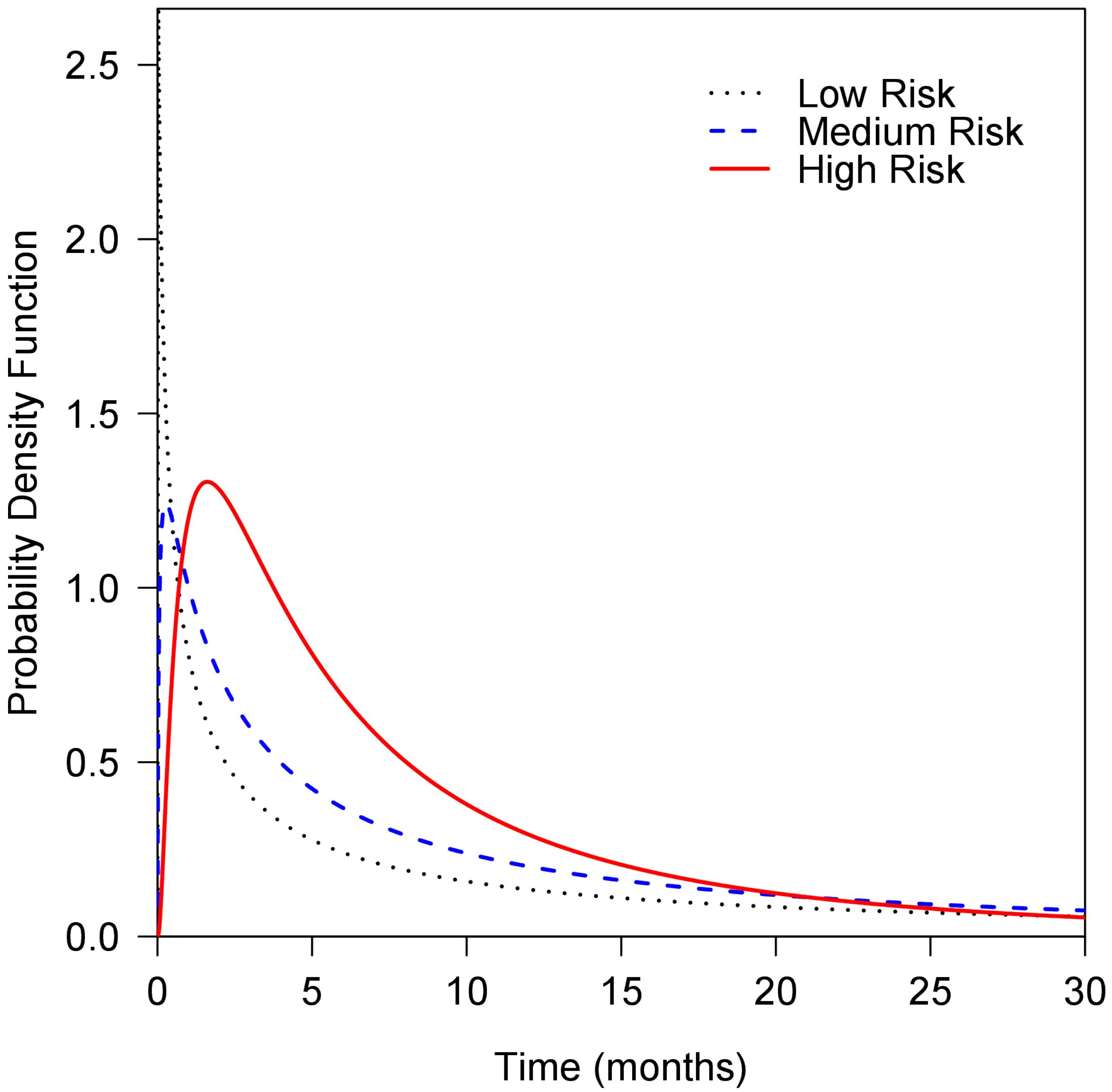

Fig. A: The lognormal probability density functions for the time to recidivism for the three risk categories.

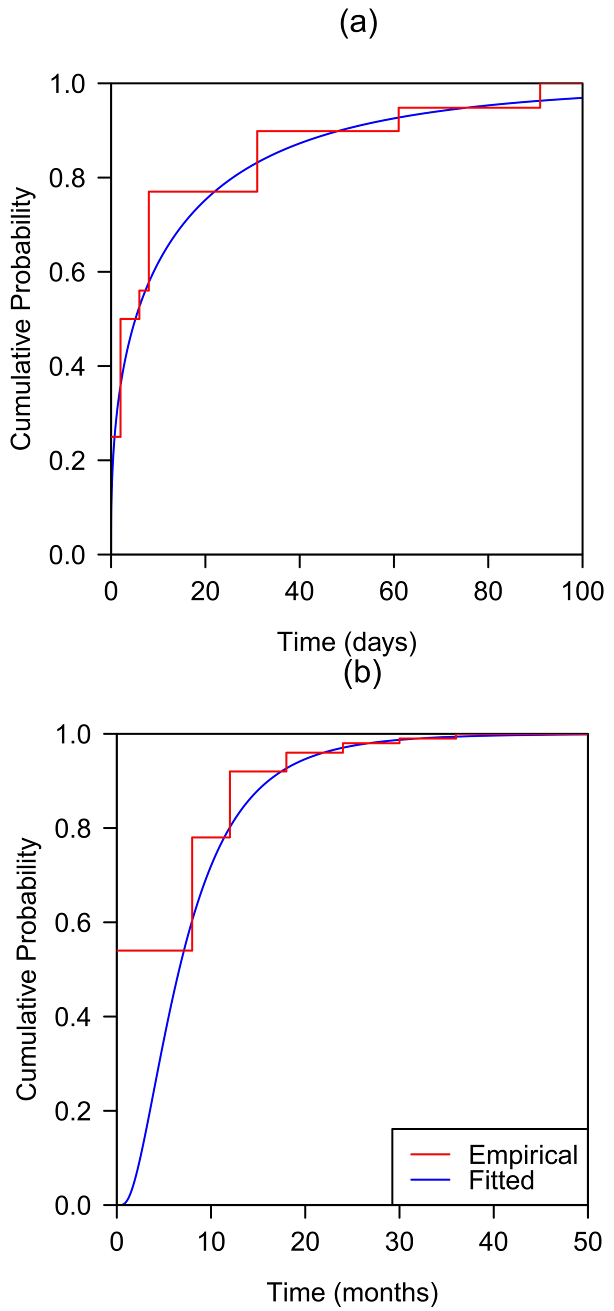

Fig. B: The empirical and fitted cumulative distribution functions for the length of post-sentence jail terms for **(a)** non-felonies and **(b)** felonies.

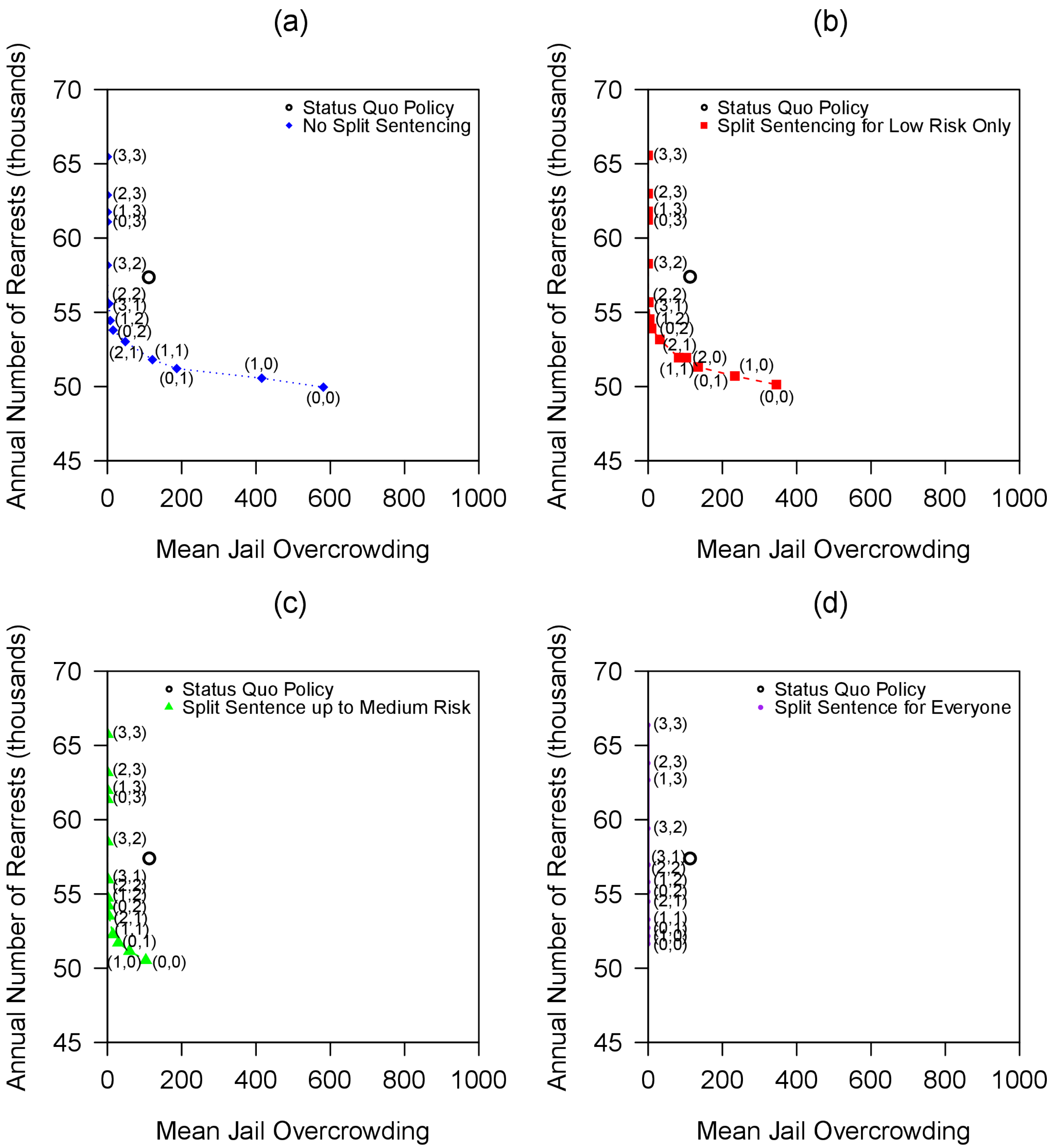

Fig. C: For each of the four tradeoff curves in Fig. B(b) in the main text, the optimal policy along the different points on the curve. Each policy is denoted by a pair of numbers, where the first number corresponds to the pretrial release for non-felonies (left column of Table A in the main text) and the second number corresponds to the pretrial release for felonies (middle column of Table A in the main text).

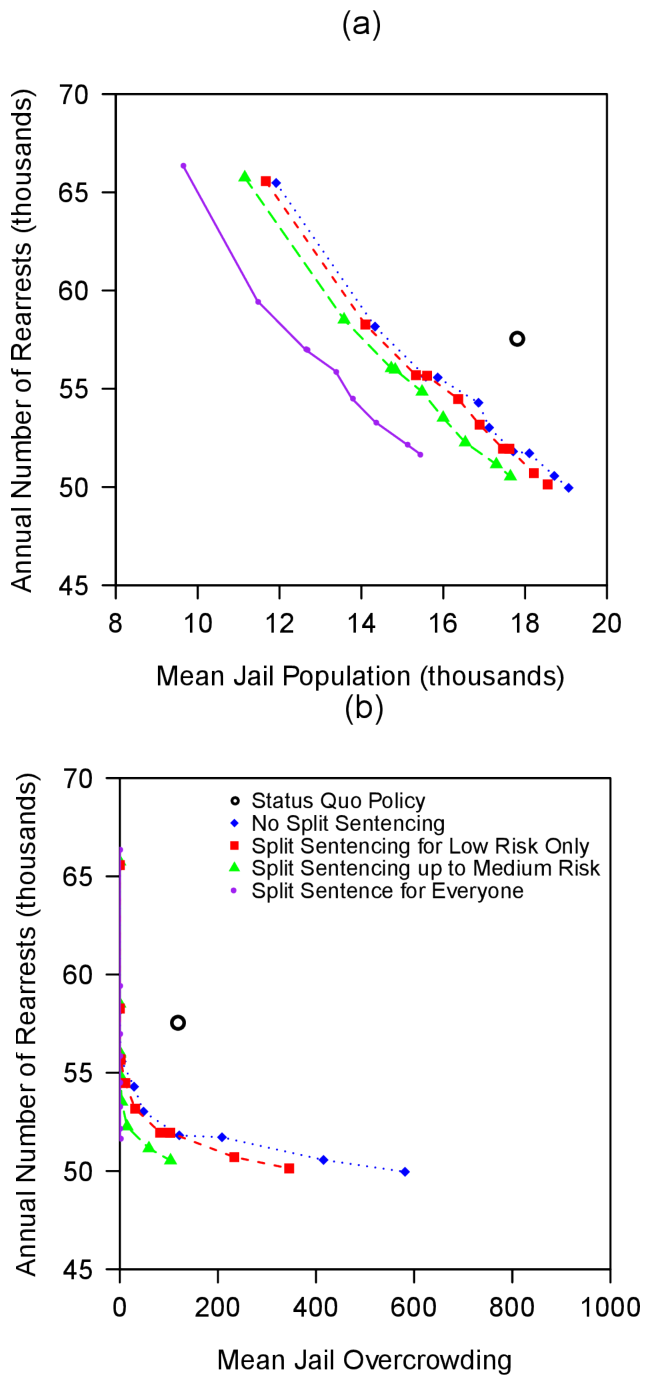

Fig. D: For each of the four options for split sentencing in the right column of Table A, the optimal (i.e., optimizing over the remaining 16 options in Table A) tradeoff curves of the annual rearrest rate vs. **(a)** the mean jail population and **(b)** mean jail overcrowding, restricting to policies that treat felonies at least as strictly (with respect to pretrial release) as non-felonies of the same risk category. The circle denotes the status quo policy for LA County in early 2014.

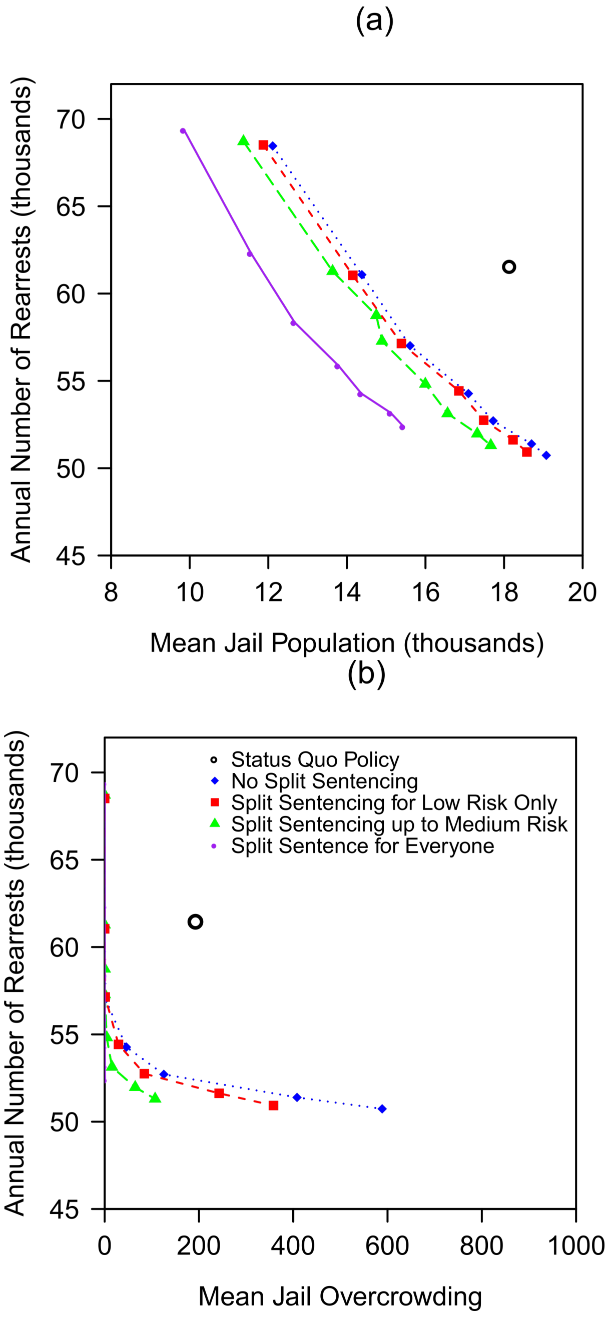

Fig. E: Sensitivity analysis of our main results with failure-to-appear probabilities changed to 0.279 for low risk and 0.425 for medium and high risks. For each of the four options for split sentencing in the right column of Table A, the optimal (i.e., optimizing over the remaining 16 options in Table A) tradeoff curves of the annual rearrest rate vs. **(a)** the mean jail population and **(b)** mean jail overcrowding, restricting to policies that treat felonies at least as strictly (with respect to pretrial release) as non-felonies of the same risk category. The circle denotes the status quo policy for LA County in early 2014.
